# Aspects of T Cell-Mediated Immunity Induced in Mice by a DNA Vaccine Based on the Dengue-NS1 Antigen after Challenge by the Intracerebral Route

**DOI:** 10.1371/journal.pone.0163240

**Published:** 2016-09-15

**Authors:** Edson R. A. Oliveira, Antônio J. S. Gonçalves, Simone M. Costa, Adriana S. Azevedo, Marcio Mantuano-Barradas, Ana Cristina M. A. Nogueira, Ada M. B. Alves

**Affiliations:** 1 Laboratory of Biotechnology and Physiology of Viral Infections, Oswaldo Cruz Institute, Oswaldo Cruz Foundation, Rio de Janeiro, Brazil; 2 Laboratory of Clinical Immunology, Oswaldo Cruz Institute, Oswaldo Cruz Foundation, Rio de Janeiro, Brazil; Animal Health and Veterinary Laboratories Agency, UNITED KINGDOM

## Abstract

Dengue disease has emerged as a major public health issue across tropical and subtropical countries. Infections caused by dengue virus (DENV) can evolve to life-threatening forms, resulting in about 20,000 deaths every year worldwide. Several animal models have been described concerning pre-clinical stages in vaccine development against dengue, each of them presenting limitations and advantages. Among these models, a traditional approach is the inoculation of a mouse-brain adapted DENV variant in immunocompetent animals by the intracerebral (i.c.) route. Despite the historical usage and relevance of this model for vaccine testing, little is known about the mechanisms by which the protection is developed upon vaccination. To cover this topic, a DNA vaccine based on the DENV non-structural protein 1 (pcTPANS1) was considered and investigations were focused on the induced T cell-mediated immunity against i.c.-DENV infection. Immunophenotyping assays by flow cytometry revealed that immunization with pcTPANS1 promotes a sustained T cell activation in spleen of i.c.-infected mice. Moreover, we found that the downregulation of CD45RB on T cells, as an indicator of cell activation, correlated with absence of morbidity upon virus challenge. Adoptive transfer procedures supported by CFSE-labeled cell tracking showed that NS1-specific T cells induced by vaccination, proliferate and migrate to peripheral organs of infected mice, such as the liver. Additionally, in late stages of infection (from the 7^th^ day onwards), vaccinated mice also presented reduced levels of circulating IFN-γ and IL-12p70 in comparison to non-vaccinated animals. In conclusion, this work presented new aspects about the T cell-mediated immunity concerning DNA vaccination with pcTPANS1 and the i.c. infection model. These insights can be explored in further studies of anti-dengue vaccine efficacy.

## Introduction

In the past two decades, dengue has appeared as the most occurring arthropod-borne illness worldwide. From a general picture of its epidemiology, it is estimated that 390 million infections occur each year, of which near a quarter is characterized with symptoms [1]. Following infection, dengue disease manifests as an array of clinical signs that varies from a non-specific febrile illness, known as dengue fever (DF), to life-threatening forms, addressed as dengue hemorrhagic fever (DHF) and dengue shock syndrome (DSS) [2]. *Aedes* mosquitoes (mainly *Ae*. *aegypti* and *Ae*. *albopictus*), found along territories of tropical and subtropical climates, transmit the etiologic agent, dengue virus (DENV), to humans [1, 3].

Structurally, DENV is a single-stranded RNA virus with approximately 50 nm of diameter that belongs to the *Flavivirus* genus from *Flaviviridae* family. It has four distinct but closely related serotypes (DENV1-4) and its genome codes for 10 viral proteins: three structural (C, prM and E) and seven non-structural proteins (NS1, NS2A, NS2B, NS3, NS4A, NS4B, and NS5) [4].

Despite the major health burden caused by DENV, no highly effective vaccine or specific therapeutic intervention has yet become available. Consequently, attempts at reducing the disease spread happens nowadays in a supportive-measure basis, which include vector control, symptomatic treatment and educational programs. Due to the inefficiency of these measures in preventing epidemics and outbreaks, the need for a specific approach against this infection is even more highlighted.

Apart from intrinsic difficulties in understanding the nature of DENV infection, another great obstacle for vaccine development against the disease is the lack of an appropriate animal model capable of mimicking the disease spectrum as observed in humans. A traditional immunocompetent mouse approach is based on the intracerebral (i.c.) inoculation of a mouse-brain adapted DENV [5]. Despite the limitations of this mouse model, regarding the route of infection and the clinical outcome, infection could induce systemic effects in the host as described elsewhere[6]. Besides, virus challenge in this mouse approach is normally lethal, hence providing a straightforward readout parameter for vaccine testing.

DNA vaccines are able to promote long-lasting cellular immunity against some pathogens, including flaviviruses [7]. Moreover, this vaccination approach promotes *in vivo* expression of antigens, mimicking what originally occurs during the course of natural viral infections. Ultimately, DNA vaccines lead to appropriate post-translational modifications and proper protein folding, features that can directly influence the quality of the elicited immunity [8, 9]. Over the last decade, our group has demonstrated the efficacy of a number of anti-dengue DNA vaccine candidates based on the i.c.-infection mouse approach [10–13]. Regarding one of these candidates (pcTPANS1, which is a vaccine based on the NS1 antigen), we found that it was able to promote high degree of protection, resulting in near 100% of survival rates upon lethal DENV challenge [10]. Recently, in an attempt to determine possible correlates of protection induced by pcTPANS1, we found that the cooperation between T cells and humoral immunity is critical for the establishment of this defense [14].

Despite these findings, the immune mechanisms involved in the protection conferred by pcTPANS1 vaccine remain elusive. For this reason and by the fact that DNA vaccination predominantly yields a sustained cell-mediated immunity [15], in the present work we aimed to investigate the T cell activation induced by vaccination on the course of i.c.-DENV infection. Flow cytometry immunophenotyping experiments revealed that pcTPANS1 induced a sustained T cell activation in spleen of DENV-infected mice. Additionally, we found that the reduced expression of CD45RB on the surface of CD4^+^ and CD8^+^ T cells correlates with the absence of morbidity upon i.c. challenge. Adoptive transfer procedures supported by CFSE-labeled cell tracking showed that NS1-specific T cells induced by vaccination, proliferate and migrate to the liver of infected mice. Regarding circulating cytokines, cytometric bead array technique (CBA) revealed that pcTPANS1-vaccinated mice also presented reduced levels of IFN-γ and IL-12p70 in comparison to non-vaccinated animals. These findings clearly exhibited that classical points of T cell-mediated immunity, such as activation, proliferation and migration that occur peripherally, are induced in pcTPANS1-vaccinated mice upon i.c. infection. Interestingly, these events occurred even in light of the immune privilege feature of the brain. After all, this work revealed new aspects about the T cell-mediated immunity concerning DNA vaccination with pcTPANS1 and the i.c. infection model.

## Materials and Methods

### DNA vaccine

A previously described DNA vaccine, pcTPANS1 [10,11], was used for immunizations. Briefly, this plasmid, derived from pcDNA3 (Invitrogen, USA), encodes the full length NS1 gene from DENV2, strain New Guinea C, fused to the human tissue plasminogen activator signal sequence (t-PA). For control purposes, we used the pcTPA plasmid without the NS1 gene. Plasmids were isolated from transformed *Escherichia coli*, DH5-α strain, and purified by Qiagen Endofree Plasmid Giga Kit (Qiagen, Germany) following manufacturer's instruction. Purified plasmids, eluted in endotoxin-free sterile water, were kept at -20°C until use. Digestion with restriction enzymes and electrophoresis were performed with the obtained DNA for quality control procedures.

### Immunization and virus challenge

**Ethics statement:** All experiments with mice undergone the process of consent according to the ethical principles in animal experimentation stated in the Brazilian College of Animal Experimentation. Ethics committee that approved this study: Comissão Ética no Uso de Animais do Instituto Oswaldo Cruz, Fundação Oswaldo Cruz (approval ID: L067/08 and LW14/12).

#### Immunization protocol

Wild-type specific pathogen free (SPF) BALB/c mice, male 4 to 6 week-old, were purchased from the Multidisciplinary Center for Biological Investigations (CEMIB, UNICAMP-SP). Animals were inoculated by the intramuscular (i.m.) route with 50 μg of plasmids diluted in 50 μL of phosphate buffer saline (PBS) in each tibialis posterior muscles (100 μg/mice), using 27-gauge needles. Each animal group received two doses of the recombinant plasmid, pcTPANS1, or control vector, pcTPA, given 2 weeks apart.

#### Mice infection

The mouse-brain adapted dengue 2 virus (DENV2), strain New Guinea C (NGC) (GenBank M29095), was used for animal infections. After passages in the brain of new born mice, the DENV2 NGC strain propagation was carried out in Vero cells cultured in 199 medium with Earle salts (E199) buffered with sodium bicarbonate (Sigma, USA) and supplemented with 10% fetal bovine serum (FBS, Invitrogen, USA). Two weeks after the second dose of the DNA immunization, BALB/c mice were anesthetized with a mixture of ketamine-xylazine [16] and intracerebrally inoculated with 30 μL of DENV2 NGC 40 LD_50_, diluted in E199 medium. For recording mortality rates, groups of animals were followed up for 21 days post infection, after which they were sacrificed. Morbidity signs were attributed as the appearance of hind-leg paralysis and alterations in spinal column. Infected or control animals were also sacrificed at different time points for kinetic studies. Samples as blood (collected by cardiac puncture), serum, spleens and livers were collected at different time points for further analysis.

#### Animal housing and method of euthanasia

Mice were grouped in microisolators (maximum of 5 per environment) with pine wood shavings and kept under dark/light cycles with 12 h of interval. The environment was under a HEPA-filtered air supply system and also provided with water and ration *ad libitum* both sterile. Mice were monitored two times per day with approximately 8 h of interval between inspections. Animals that presented CNS-related symptoms upon virus challenge, such as hunchback posture with hind-leg paralysis, were readily euthanized. Under our experimental procedure, mice usually start to present CNS-related morbidity in a window between the 7^th^ and the 11^th^ dpi (day post infection). No animal died before the referred window. Euthanasia was carried out by the administration of a dissociative agent combination (ketamine/xylazine) in high dose followed by exsanguination [17–19], and all efforts were made to minimize suffering.

### Immunophenotyping

Flow cytometry technique was employed for immunophenotyping leukocytes isolated from blood, spleen and liver samples. The organs were dissociated in wire mesh screens using RPMI medium. Spleen macerates and blood samples were treated with BD FACS Lysing for red blood cell lysis and fixation according to manufacturer's instructions (BD Biosciences, USA). Liver-infiltrated leukocytes were isolated by Ficoll gradient. In this case, liver macerates were gently transferred to tubes containing 5 mL of Ficoll. Samples were spinned down at 800 g for 30 min at room temperature. The mononuclear cell ring and interphase were isolated, washed two times and suspended in PBS pH 7.4. Samples were then treated with BD FACS Lysing solution. Isolated cells were finally washed and suspended in PBS/BSA 1%. Approximately 10^6^ cells were stained on ice for 20 min in the dark with the following mAb combination: CD3-PE, CD4-Alexa Fluor 647, CD8-PerCP and CD45RB-FITC. All mAbs used for this work were obtained from BD Biosciences and background-staining controls were performed using isotypes recommended by the manufacturer. Samples were read in a BD FACS Canto II and analyzed offline with FlowJo (ThreeStar Inc, USA) software.

### T cell enrichment, carboxyfluorescein succinimidyl ester (CFSE) staining and adoptive cell transfer

#### Preparation of T cell-enriched suspensions

For cell transference experiments, spleens were collected from donors (BALB/c mice immunized or not with pcTPANS1, n = 13 per group), obtained two weeks after the second DNA dose (for the immunized group) and without virus challenge. At this same period, serum samples were also collected from donors. Spleens were disrupted using wire mesh screens and splenocytes were isolated in RPMI-1640, containing 1% penicillin/streptomycin (10,000 U/mL, Invitrogen) and supplemented with 5% FBS. Cells were incubated in culture medium for 1 h, and then, for another hour in nylon wool column (previously packed and stabilized with RPMI in 5% CO2 atmosphere), both at 37°C. After elution from nylon wool column with RPMI medium, in order to remove B lymphocytes, the resultant T cell-enriched suspension was then suspended in RPMI-1640 medium and adjusted to a final concentration of 3 x 10^7^ cells/mL. In order to evaluate the enrichment protocol, samples were collected before and after the nylon wool purification procedure and then stained with anti-CD3-PE, anti-CD4-FITC and anti-CD8-PerCP for flow cytometry analysis.

#### CFSE staining

Approximately 3 x 10^8^ splenocytes obtained from donors (vaccinated or naïve mice) after the T cell enrichment procedure were incubated with CFSE 8.5 μM (CellTrace, Invitrogen) in PBS for 15 min at 37°C. After incubation, cells were washed and suspended in RPMI medium without FBS and then adjusted to a concentration of 2 x 10^7^ cells/100 μL. In order to evaluate the efficiency of this protocol, samples were collected after the procedure and then stained with, anti-CD4-Alexa Fluor 647 and anti-CD8-PerCP for flow cytometry analysis. For adoptive cell transfer, 100 μL of T cell-enriched suspension labeled with CFSE (containing around 2 x 10^7^ cells) were used. Cells (purified from vaccinated or naïve mice) were intravenously injected in recipient mice (n = 6 per group) by the retro-orbital route. Injections were performed 4 days after virus challenge. Animals also received 1 mL of serum (collected from vaccinated or naïve donors) by the intraperitoneal (i.p.) route. After 3 days, recipient mice were sacrificed and blood, spleens and livers were sampled for flow cytometry analysis. Isolated cells from each site of analysis were pooled and stained with anti-CD4 and anti-CD8. Samples were read in a BD FACS Canto II and analyzed offline with FlowJo (ThreeStar Inc, USA) software.

### Cytokine quantification

Cytokines were measured with the multiplex BD CBA Mouse inflammation kit (BD Bioscience) using plasma samples. In this analysis, interleukin-12p70 (IL-12p70), tumor necrosis factor-α (TNF-α), interferon-γ (IFN-γ), monocyte chemotactic protein 1 (MCP-1), interleukin-10 (IL-10) and interleukin-6 (IL-6) were simultaneously quantified in each sample. Detections were performed according to the manufacturer's instructions with modifications. Briefly, beads coated with each of the six specific capture antibodies were pooled. Subsequently, 25 μL of the mixed captured beads, 25 μL of the tested plasma sample or the provided standard cytokines and 25 μL of PE-detection reagent were added consecutively to each assay tube and incubated for 2 h at room temperature in the dark. Samples were washed and centrifuged at 200 g for 5 min. Supernatants were discharged and the bead pellets were suspended in 300 μL of a buffer provided by the manufacturer. Samples were read on a BD FACS Canto II Flow Cytometer and analyzed by FCAP Array^™^ Software (BD Bioscience). Cytokine standards were serially diluted for the construction of calibration curves to assess cytokine concentrations in tested samples. The theoretical limits of detection were 10.7 pg/mL for IL-12p70, 7.3 pg/mL for TNF-α, 2.5 pg/mL for IFN-γ, 52.7 pg/mL for MCP-1, 17.5 pg/mL for IL-10 and 5.0 pg/mL for IL-6 (S1 Fig).

### Statistics

Data were analyzed with GraphPad prism software v5.1 (La Jolla, USA) using non-parametric statistical tests. Significant differences between analyzed groups (immunophenotyping and cytokine quantification data) were determined using Mann-Whitney test with *p < 0.05, **p < 0.01 and ***p < 0.001. Regarding the cytokine quantification, a regression curve for each serially diluted standard cytokine was obtained considering the 5-parameter logistic model (5PLM) using the FCAP Array^™^ Software (BD Bioscience) v3.0.

## Results and Discussion

### Time course of T cell activation in pcTPANS1-vaccinated mice upon i.c.-DENV infection

The protective properties of pcTPANS1 in mice, as determined by increased survival rates and reduction of morbidity, were already demonstrated in previous works [10,11]. Recently, we have also shown the importance of T cells, mainly CD4^+^, and antibodies against the NS1 in the protection induced by such vaccine [14]. In the present work, aiming to investigate the time-course of the T cell activation in vaccinated mice after virus challenge, a kinetic study was performed considering pcTPANS1-immunized animals in comparison to pcTPA-inoculated and naïve groups (controls). Following the standard vaccination/challenge protocol, we immunized the groups of mice with two DNA doses and challenged two weeks after the second dose. Days 0, 1, 3, 5, 7 and 8–11 post infection (dpi) were considered for evaluations. At each evaluation point/period, blood and spleens were sampled for flow-cytometry immunophenotyping and T cell activation was assessed by the reduced expression of CD45RB (CD45RB^low^) on the surface of CD3^+^CD4^+^ and CD3^+^CD8^+^ cells.

**Fig 1:**
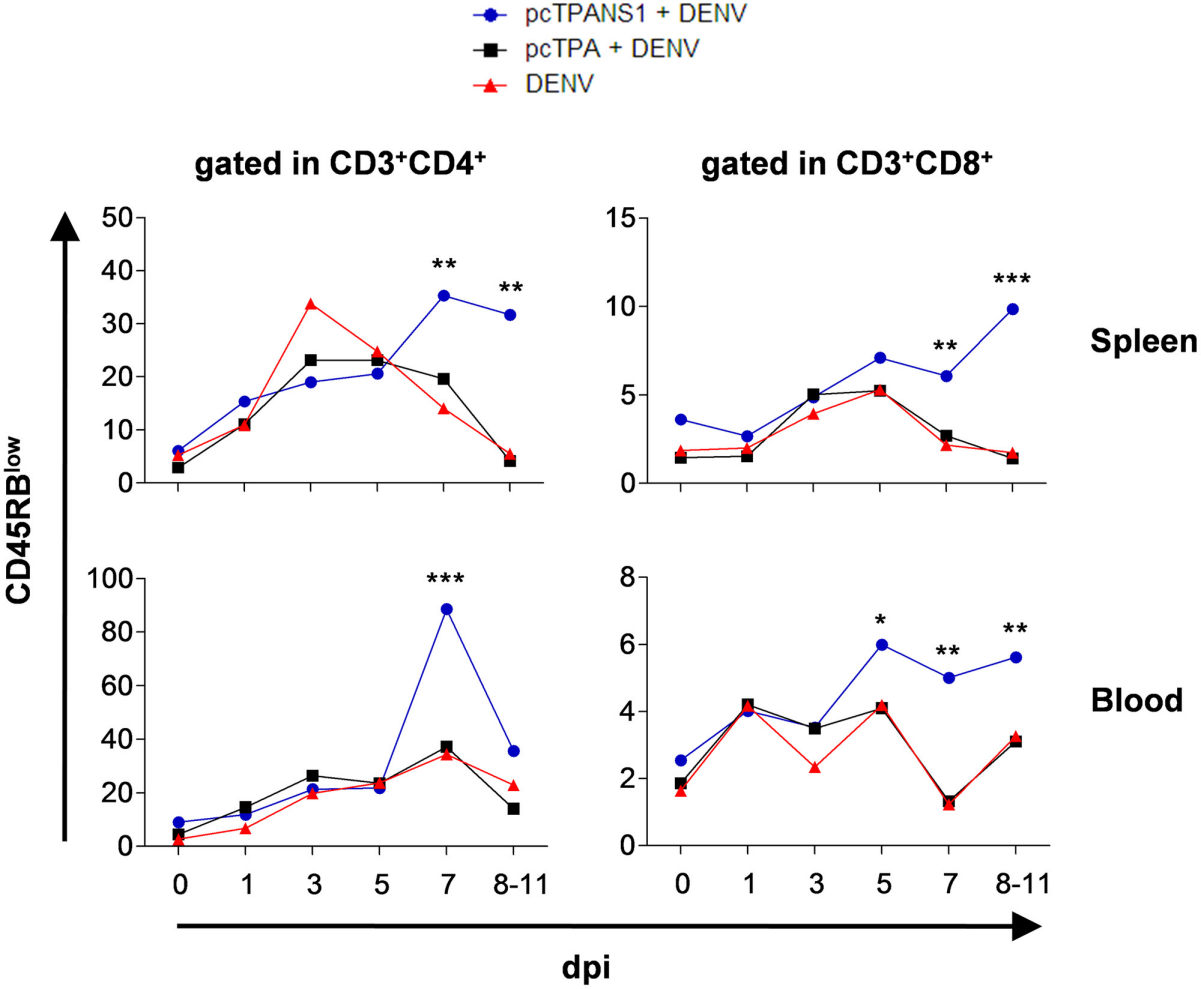
T Cell activation in spleen and blood of DENV-infected BALB/c mice immunized with pcTPANS1. BALB/c animals (minimum of 5 per group) were immunized with pcTPANS1 and then challenged with DENV2. To control the experiment, naïve mice or animals that received the pcTPA plasmid were also challenged. Spleen and blood samples were collected at days 0, 1, 3, 5, 7 e 8–11 post infection (dpi) and analyzed by flow cytometry using anti-CD3, anti-CD4, anti-CD8 and anti-CD45RB surface cell markers. Values are expressed as median of the percentage of cell subpopulations. Significant differences between groups at the same day of the kinetic study were evaluated considering the non-parametric Mann-Whitney statistical test (*p < 0.05; **p < 0.01; and ***p < 0.001). Data are representative of three independent experiments.

Considering the non-vaccinated groups (pcTPA-inoculated and naïve animals) after virus infection, the variations of the percentage of activated T cells in the spleen behaved similarly throughout the kinetic study. We observed that CD4^+^ or CD8^+^ T cell percentages peaked between the 3^rd^ and 5^th^ dpi, after which they dramatically dropped reassuming the basal initial values. In the vaccinated group, the percentage of activated T cells in spleen showed a similar pattern of increase until the 5^th^ dpi, when compared to controls. However, after this point, levels of activated T cells kept increasing in pcTPANS1-immunized animals until the end of the kinetic study (Fig 1 top).

Regarding the analysis of blood samples, non-vaccinated groups exhibited a discrete increase in the percentage of activated CD4^+^ T cells along the five initial days of infection, with a peak at the 7^th^ dpi. Vaccination induced a similar behavior in levels of activated CD4^+^ T cells until the 5^th^ dpi, when compared to controls, while afterwards a 3-fold higher increment prompted at the 7^th^ dpi. Higher percentages of activated CD8^+^ T cells, in comparison to controls, were also found in blood of vaccinated mice, with significant differences detected since the 5^th^ dpi, after which levels were kept higher until the end of the kinetic study (8–11 dpi) (Fig 1 bottom).

These data suggested that DENV-NS1 antigen, which the pcTPANS1 DNA vaccine mediated the expression, was able to induce a clear adaptive T cell-mediated immunity. Following virus challenge, the T cell induction characterized a sustained activation in secondary lymphoid organs. Since the role of T cells during viral infections is well described [20, 21], an array of mechanisms including T cell memory, direct cytotoxicity and production of pro-inflammatory cytokines is potentially feasible under this experimental condition. As we found activated T cells in the circulation of vaccinated-challenged mice, data also suggested a migration of cells displaying effector phenotypes to the periphery. Along with the clear induction of activation and with the protective properties of pcTPANS1 against the infection, the reduced expression of CD45RB also suggested a considerable vaccine-induced T cell memory phenotype. However, other cell surface markers would still be necessary for a more consistent description regarding this kind of cell differentiation.

Based on the analyzed spleen and blood samples, vaccination with NS1 induced a strong CD4^+^ T cell response rather than CD8^+^, evidenced at the 7^th^ dpi. At this time point, the difference in percentages between activated CD4^+^ and CD8^+^ T cells were about 30% vs 5% in spleen and 80% vs 5% in blood, respectively. These findings were in line with a previous report from our laboratory, in which the CD4^+^ response induced by this NS1-based DNA vaccine was critical for protection, as shown by depletion and passive transference experiments [14]. In the same line of thought, it was found in humans that CD4^+^ T cell epitopes preferentially target the NS1 protein while CD8^+^ epitopes were more related to the recognition of other antigens such as NS3 and NS5 [22]. Regardless of these reports that supported our findings, non-vaccinated infected mice presented higher CD4^+^ T cell response when compared to CD8^+^, what is not found in human WT-DENV infections. One hypothesis to explain this occurrence is that differences concerning the host immunity (human vs mice), virus strains (mouse-brain-adapted vs WT viruses) and inoculation route (intracerebral vs subcutaneous) would eventually favor a distinct scenario in these cellular responses.

By this point, an important question that arises is how vaccination was able to trigger classical immune responses upon the introduction of antigen in the CNS, which is widely known as an organ protected by a physical blood-brain barrier (BBB) [23]. Even considering the immune privilege properties of the CNS, apparently, a communication between the brain and the periphery exists. In this case, T cells may be involved in alternative ways to enter the CNS [24–27] and virus particles possibly spread to the circulation because of the commitment of regular tissue structures. These concerns are in congruence with our recent report in which i.c.-infected BALB/c mice presented a discrete virus spread to the circulation [6]. A direct evidence of this dissemination was the detection of DENV-NS3 antigen in the liver of I.c.-infected animals, which denoted *in situ* virus replication [6]. Thus, it would be reasonable to suggest that effector T cells derived from immunizations could be targeting infected peripheral organs, contributing, at least in part, for the overall protective role yielded by vaccination. In line with this assumption, other studies with mice have shown a critical role for T cells in protection against DENV following vaccination [28]. Tests with mice lacking the alpha/beta interferon receptor (IFN-α/β R^-/-^) indicated the protective role of CD4^+^ T cells, which particularly contributed significantly to viral clearance when induced by immunization [29]. Additionally, as reported elsewhere, CD8^+^ T cells modulated anti-DENV responses in humans [30–33].

### Levels of T cell activation and circulating cytokines versus CNS-related clinical signs after i.c. infection

In the previous analysis, we investigated the influence of pcTPANS1 vaccination over T cell activation upon the i.c. infection with DENV. We next proceeded with an analytical approach aiming to correlate the infection-induced clinical manifestations with the observed levels of activated T cells. In the adopted i.c. infection model, CNS-related morbidities (hind-leg paralysis and alterations in spinal column) are usually evidenced from the 7^th^ dpi onwards. Additional immunization/challenge experiments were then conducted considering only the 7^th^ and the 8^th^-11^th^ dpi. Right before sacrifice, animals were evaluated according to the manifestation of symptoms and then grouped as “with” or “without” clinical signs of infection.

**Fig 2:**
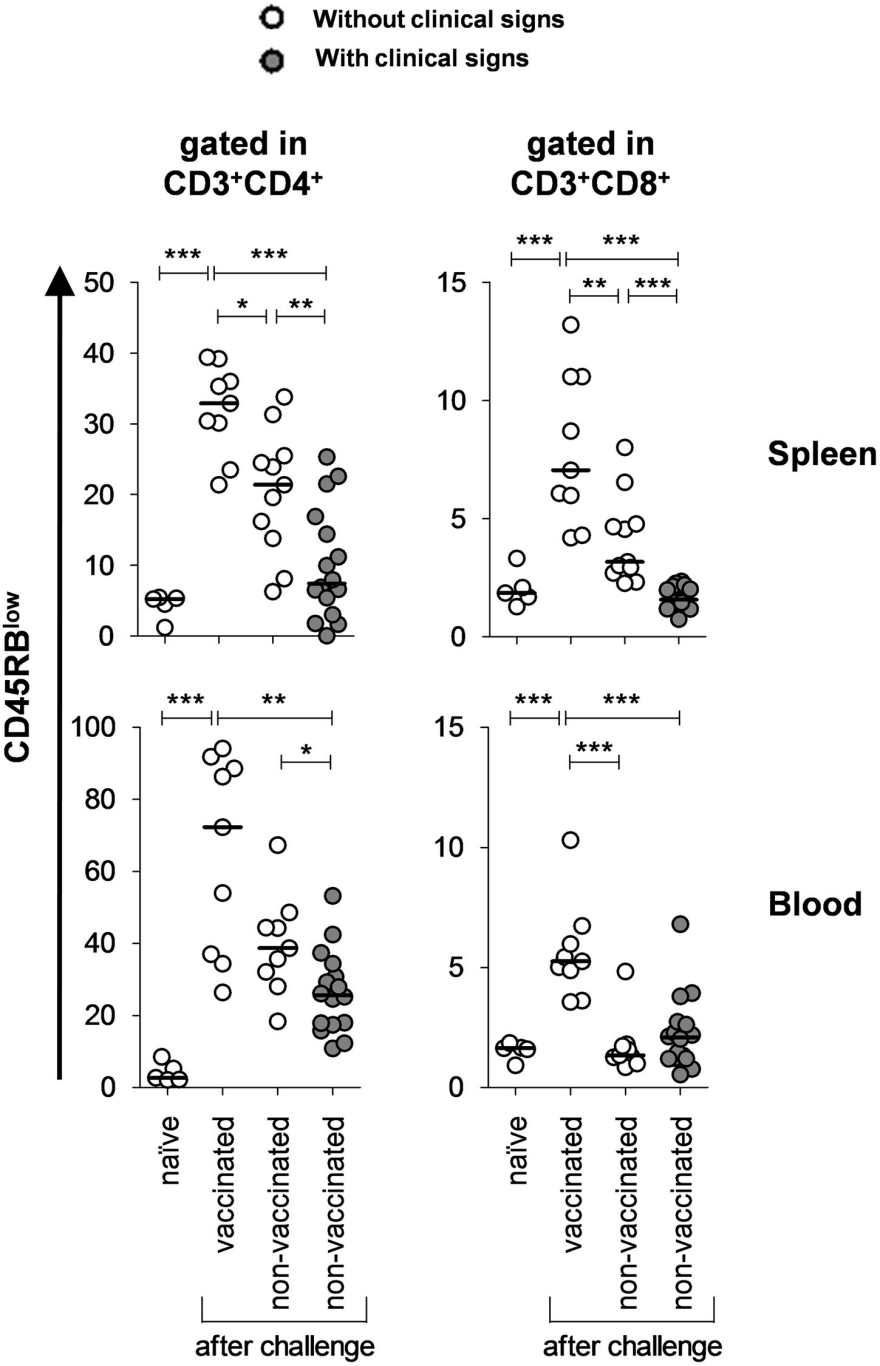
Correlation between morbidity and levels of activated T cells in spleen and blood of i.c.-challenged mice. Splenocytes as well as leukocytes from blood samples were isolated from BALB/c mice immunized or not with pcTPANS1 and i.c.-challenged with DENV2. Activated T cell phenotypes (CD3^+^CD4^+^CD45RB^low^ and CD3^+^CD8^+^CD45RB^low^) were analyzed by flow cytometry and correlated with the occurrence of clinical signs in the animal groups. Samples were considered from the 7^th^ day of infection onwards, period when clinical signs regarding the CNS commitment start to occur. Values are expressed as percentages of T cell populations with median. Significant differences between groups were evaluated using the non-parametric Mann-Whitney test (*p < 0.05; **p < 0.01; and ***p < 0.001). Data are representative of two independent experiments (naïve n = 5; vaccinated n = 9; non-vaccinated and without clinical signs n = 11; non-vaccinated and with clinical signs n = 16).

Levels of activated CD4^+^ and CD8^+^ T cells were significantly higher in spleen and blood samples of mice without the symptoms of infection due to vaccination, in comparison to non-vaccinated animals exhibiting CNS-related symptoms. In this case, increments were from 2- up to 4-fold, what demonstrated a clear association between increased levels of CD45RB^low^ T cells and the prevention of morbidity under these experimental conditions. Another observation was that among non-vaccinated mice, animals that did not show clinical signs after infection presented, in general, higher percentages of CD45RB^low^ T cells in blood and spleen (Fig 2).

The CD45RB cell marker is a member of protein tyrosine phosphatase family expressed on leukocytes and known as an essential regulator in T lymphocytes [34]. From a general picture of our findings, we could suggest that the surface expression of CD45RB^low^ on T cells could be linked to the absence of morbidity after infection in this mouse approach. In line with this suggestion, adoptive transfer experiments have shown the dichotomic behavior of CD4^+^ T cells considering the level of CD45RB expression. In SCID mice, CD4^+^CD45RB^high^ phenotype (naïve T cells) was correlated with the induction of inflammation while CD4^+^CD45RB^low^ T cells (activated/memory T cells) could prevent its development [35]. Another possibility is the activation of regulatory T cells (T_reg_ cells). In fact, CD4^+^CD45RB^low^ T cell population contains CD25^+^Foxp3^+^ T_reg_ cells, which are responsible for the regulatory activity of this T-cell subset [36]. Moreover, targeting the CD45 tyrosine phosphatase with a tolerogenic anti-CD45RB mAb acutely increases T_reg_ cell numbers in WT mice, as shown elsewhere [37]. Regarding CD8^+^ T cells, it has been shown that the CD45RB status acts as an affinity-based differentiation switch to yield potent secondary effectors following re-challenge [38].

All together, the above aspects gave rise to new ideas on how T cell-mediated immunity would act during the primary infection and after vaccination with pcTPANS1, considering the i.c. infection model. We understood that CD45RB^low^ cell marker correlates with host resistance upon infection. This finding is interesting and may reveal an indicator of host attempt to circumvent the scenario, however, in most cases it seems to be eventually overwhelmed by the disease process in the absence of immunization. Hypothetically, in the primary response, peripheral-infected organs could present damage associated to the virus presence or due to become a target for CD45RB^high^ T cells, since this subpopulation circulates in higher levels in non-vaccinated groups of mice. In agreement to this hypothesis, among other peripheral effects occurring in this model of infection, it included virus detection in circulation, presence of lymphocyte infiltrates and tissue damages in the liver and brain [6]. In contrast, a second aspect of our hypothesis would be that, upon pcTPANS1 immunization, i.c.-infected mice protection occurs by a mechanism that implicate CD45RB^low^ T cells containing effector subsets.

Levels of circulating cytokines are a direct consequence of the cellular immune response and are often linked to dengue pathogenesis [39–45]. In order to investigate the impact of the present immunization/challenge protocol in the cytokine levels, we next carried out analysis considering the levels of some cytokines related to inflammation (IL-12p70, TNF-α, IFN-γ, MCP-1, IL-10 and IL-6) and the clinical outcome after i.c. infection with DENV. Due to the timing of morbidity manifestation post-infection, the period from 7^th^ to 11^th^ dpi was again considered in this approach.

**Fig 3:**
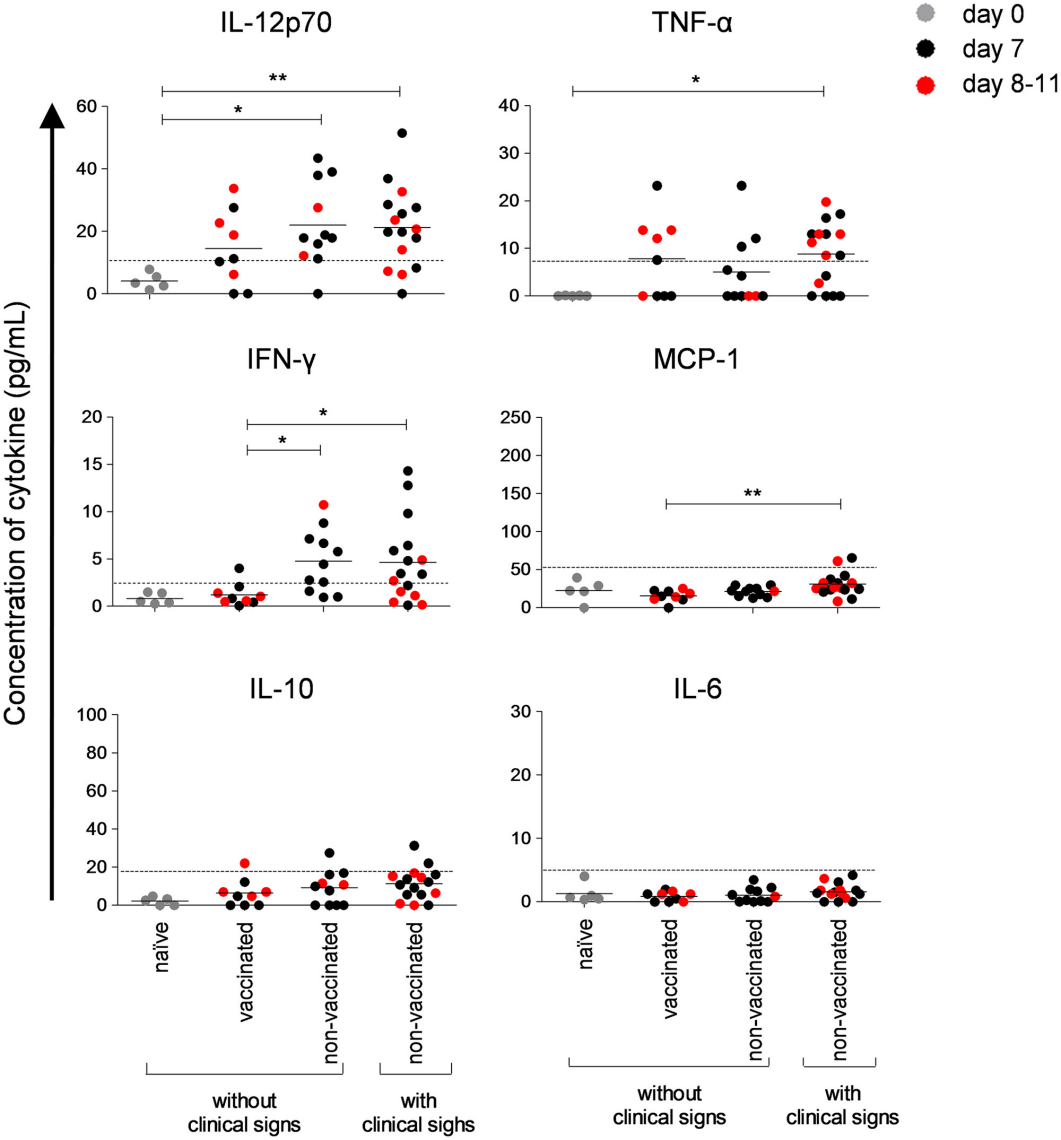
Peripheral cytokine profile in mice immunized with pcTPANS1 after challenge with DENV. Plasma samples from DENV-challenged BALB/c mice, immunized or not with pcTPANS1, were collected after the 7^th^ day of infection and tested for IL-12p70, TNF-α, IFN-γ, MCP-1, IL-10 and IL-6. The procedure was performed using the cytometric bead array (CBA) multiplex determination technique. Standardization for each cytokine is depicted in S1 Fig. Determinations are represented individually with median for each group. Dashed lines stands for the theoretical safe limits of detection. Significant differences between groups were evaluated using the non-parametric Mann-Whitney test (*p < 0.05 and **p < 0.01).

We found that non-vaccinated animals, divided as presenting or not CNS dysfunctions, did not differed significantly between each other in serum levels of any evaluated cytokine. Vaccinated mice exhibited lower amounts of circulating IFN-γ in comparison to animals that manifested clinical signs. We observed the same tendency with IL-12p70 levels; however, this discrepancy was not statistically significant (Fig 3). A hypothesis for the development of severe dengue in humans is characterized by an uncontrolled production of pro-inflammatory and vasoactive cytokines, which includes IFN-γ and IL-12p70, that would result in vascular leak [46]. In our study, the divergence in these circulating cytokine levels were not as high as found in humans. This may have occurred due to intrinsic characteristics of the chosen animal model, such as the limited systemic involvement of virus spread in function of the chosen inoculation route. As a reflex of this feature, levels of some analyzed circulating cytokines, such as MCP-1, IL-10 and IL-6, were below the theoretical limits of detection. Even so, we could detect a significant difference in levels of MCP-1 between vaccinated and non-vacinated animals presenting clinical signs.

Briefly, we envisioned new insights concerning the circulating cytokine levels and their link with the CNS-related morbidity developed post-infection. The lower levels of IFN-γ and IL-12p70 detected in protected animals suggest that the immune response elicited by vaccination in these mice occur in a more controlled fashion upon virus challenge. As we found higher numbers of activated T cells in vaccinated animals with lower levels of circulating IFN-γ, T cells may not be the major producers of this cytokine. Further studies involving the assessment of intracellular cytokine production or *in situ* staining are still necessary to allow discrimination of the major cell-type sources.

### NS1-specific CD4^+^ and CD8^+^ T cells induced by immunization migrates to the liver upon i.c. infection with DENV

**Fig 4:**
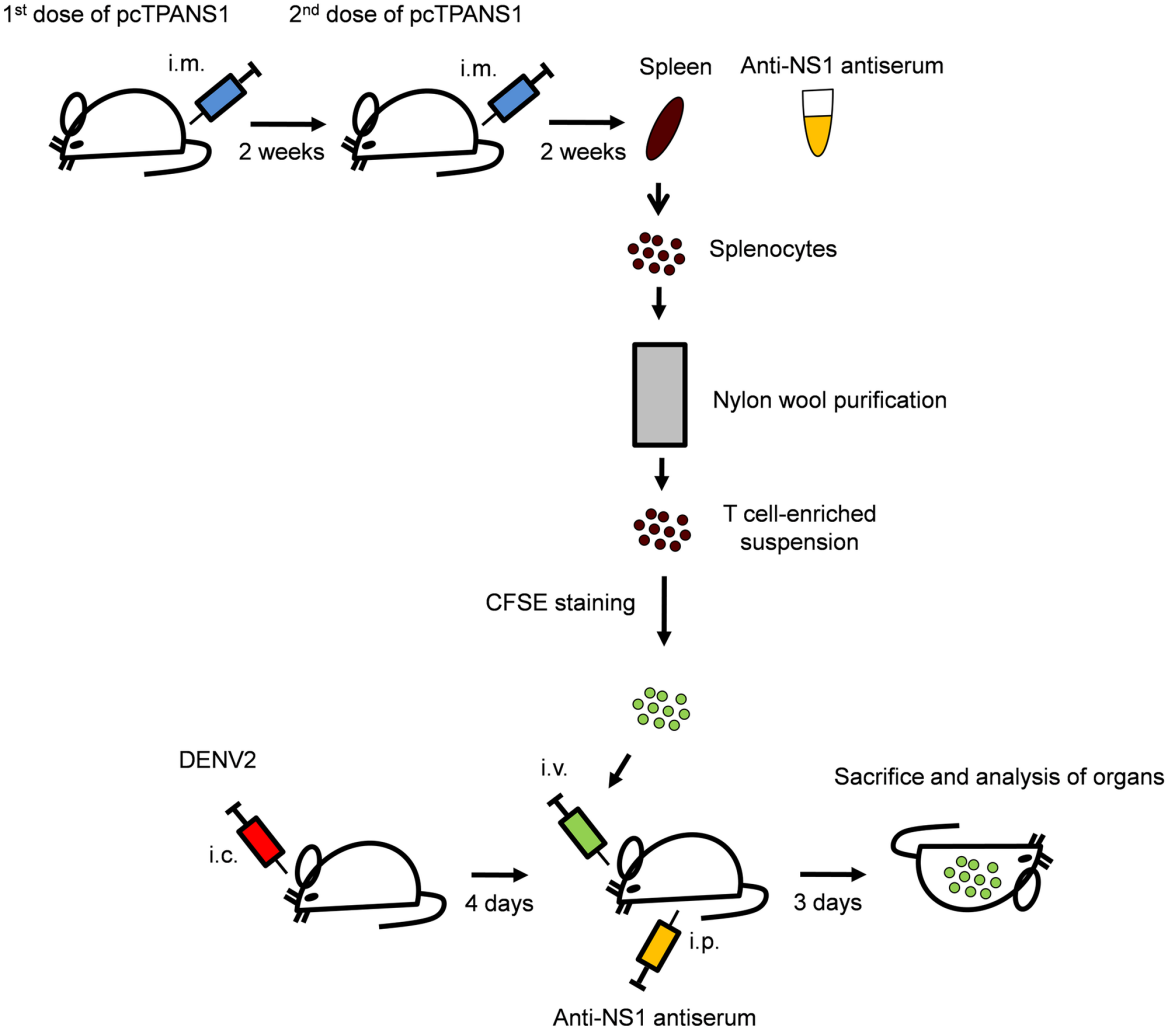
Schematic representation of T cell enrichment, CFSE staining and cell transference procedure. Groups of BALB/c mice (n = 13) received two doses of pcTPANS1 by the intramuscular (i.m.) route with two weeks of interval between each dose. Two weeks after the second dose, spleen and anti-NS1 antiserum samples were collected from these animals. T cell-enriched suspensions were obtained from isolated splenocytes by nylon wool purification and then labeled with CFSE. Cells were then transferred by the intravenous (i.v.) route to BALB/c recipients (n = 6) which were previously challenged (4 days) with DENV by the intracerebral (i.c.) route. Recipient mice also received 1 ml of anti-NS1 antiserum. After 3 days, recipients were sacrificed and spleen, blood and liver samples were analyzed by flow cytometry. To control the experiment, the same procedure was performed in parallel with splenocytes collected from non-vaccinated mice (n = 13).

At this point, we have observed a sum of characteristics in vaccinated-challenged mice that correlated with a better clinical outcome after the i.c. infection. Not only the higher amounts of activated T cells in spleen and blood, but also lower levels of circulating pro-inflammatory cytokines (IFN-γ and IL-12p70) were apparently linked to the protective properties of pcTPANS1 vaccination. Aiming to a general picture of the participation of T cells regarding activation, proliferation and migration events during the primary immunity or during the adaptive immunity, we designed a cell-transference experiment with CFSE. The schematic description of this experiment is shown in Fig 4.

Splenocytes from naïve or pcTPANS1-vaccinated mice undergone a T cell enrichment procedure and the cells obtained were stained with CFSE. This initial step of T cell enrichment processing was evaluated via flow cytometry. Increments of 84.7% of CD3^+^ cells were found in average (from 38.6% to 70.1% in the naïve group and from 37.0% to 69.5% in the pcTPANS1-immunized group, S2 Fig panels A and B). The final enriched CFSE-labeled T cell suspensions presented increments of 41.9% and 79.5% in average regarding levels of CD8^+^ and CD4^+^ cells, respectively (S2 Fig panels A and C).

Following the purifying/labeling procedure, final suspensions were i.v.-introduced in syngeneic-recipient mice at the 4^th^ day post i.c.-DENV infection. As previously described concerning this vaccination/challenge protocol, cell transference experiments revealed that T cells in combination with serum is critical for protection of mice against the i.c.-DENV infection [14]. In order to mimic this same protective environment, recipient mice also received anti-NS1 antiserum or serum collected from naïve animals, both intraperitonealy. Analyzed groups were divided as "Vac_transf_"—infected animals that received CFSE-labeled T cells and serum from pcTPANS1-vaccinated donors; and "Naïve_transf_"—infected animals that received CFSE-labeled T cells and serum from naïve donors. Three days after the cell transference (reaching the 7^th^ day of infection), recipient mice were sacrificed and blood, spleen and liver samples were considered for flow cytometry analysis. In a general view, the behavior of labeled-transferred T cells in the Naïve_transf_ animals was considered to investigate the proliferation and distribution of these cells during a primary i.c. infection. On the other hand, to study the adaptive immunity induced by pcTPANS1 vaccination followed by the i.c. challenge we investigated the same aspects concerning these labeled cells in Vac_transf_ animals.

**Fig 5:**
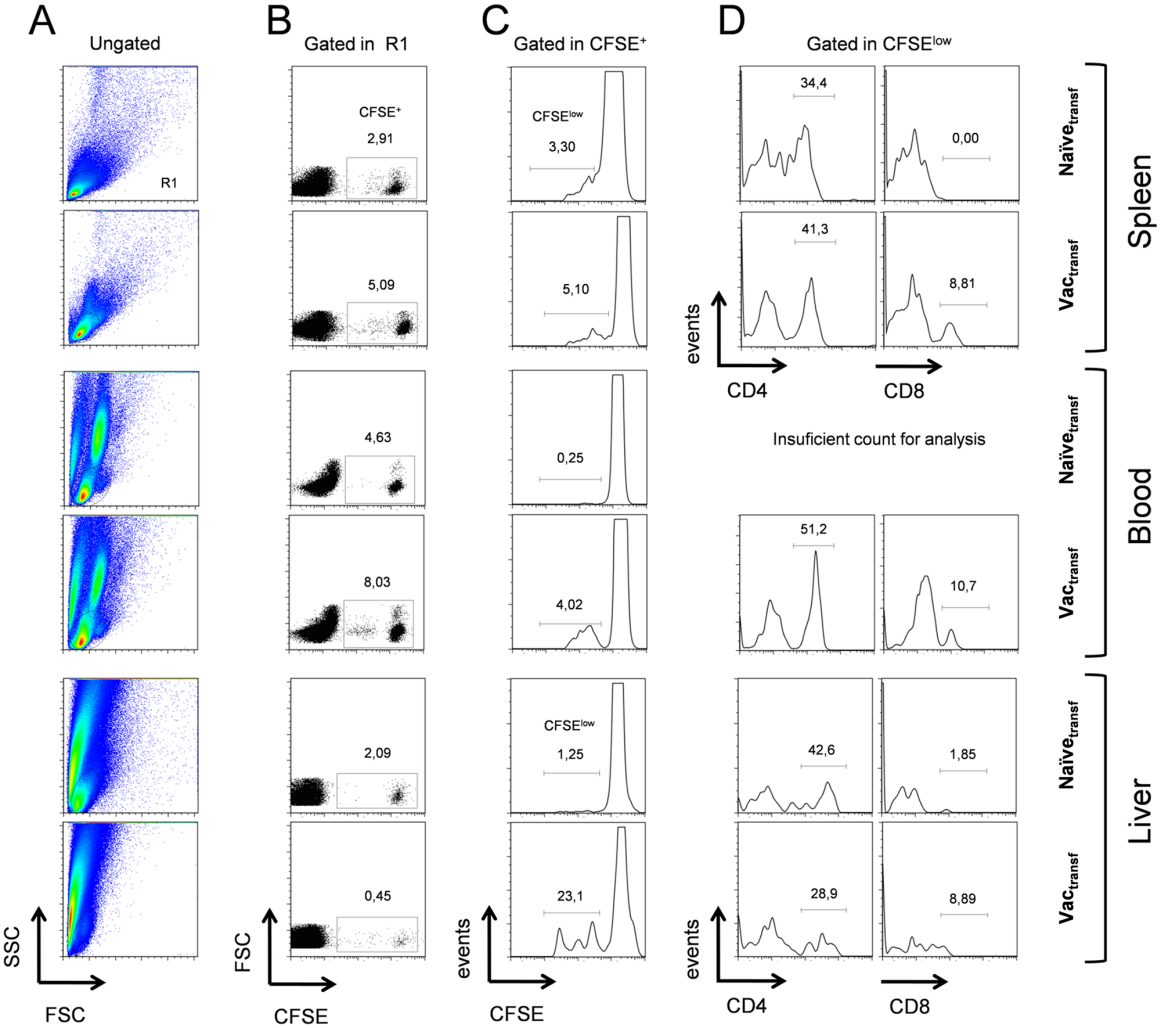
Migration and proliferation of lymphocytes due to vaccination effect on the course i.c. infection with DENV. Splenocytes obtained from naïve or pcTPANS1-vaccinated BALB/c mice (n = 5 per group) were submitted to a T cell enrichment procedure and then labeled with CFSE. In sequence, labeled cells were transferred to recipient BALB/c mice (n = 5 per group) that were previously challenged (4 days) with DENV2 by the i.c. route. Three days after the cell transference (reaching a total of seven days of infection), animals were sacrificed and spleen, blood and liver were sampled for flow cytometry analysis considering CFSE, anti-CD4 and anti-CD8 as labels. (A) Density plot representation of ungated events considering all studied samples evidencing the region of analysis (R1) created under morphologic parameters of size (FSC) and granulosity (SSC). (B) Dot plot representation of events gated in R1 exhibiting a region considered as CFSE^+^ cells that were recovered from the recipient mice. Analysis were normalized to 50,000 events regarding spleen and blood samples and to 10,000 events regarding liver samples. (C) Flow cytometry histograms gated in CFSE^+^ events showing the CFSE^low^ region (considered as the cell proliferation region). (D) Evaluation of CD4^+^ and CD8^+^ events comprised in the cell proliferation region. Vac_transf_—sample from DENV-challenged recipients that received T cell-enriched CFSE-labeled cell suspension from pcTPANS1-vaccinated mice; Naïve_transf_—sample from DENV-challenged recipients that received T cell-enriched CFSE-labeled cell suspension from non-vaccinated mice (control group).

Results showed that blood and spleen samples obtained from Vac_transf_ group presented higher percentages of recovered-CFSE^+^ cells, when compared to percentages of the same population found in the Naïve_transf_ group. In this case, we observed increments of approximately 75% in both analyzed sites (Fig 5 panel B). By this observation, higher amounts of transferred T cells from vaccinated donors appeared to be directed to different sites in recipient mice, what is consistent with classical contrasts of adaptive versus primary immunity.

Considering the recovered-CFSE^+^ cells from spleen samples of Vac_transf_ group, 5.10% were CFSE^low^, while this population represented 3.30% of the CFSE^+^ events in Naïve_transf_ animals (Fig 5 panel C top). In line with our previous results regarding activation of T cells, this data reaffirmed that in both situations (primary infection and adaptive immunity) specific clonal expansion of lymphocytes was induced upon i.c. infection. Because of the faster proliferation due to the presence of NS1-specific memory T cells transferred to recipient mice, differences between Vac_transf_ and Naïve_transf_ groups (nearly 55% higher in Vac_transf_ animals) may be due to intense proliferation of NS1-specific clones. Interestingly, we found no CD8^+^ T cells among CFSE^low^ events concerning the Naïve_transf_ animals, while this population represented nearly 9% of the CFSE_low_ region regarding the spleen of Vac_transf_ animals (Fig 5 panel D top). This data was consistent with a previous report in which authors assessed the functional activity of CD8^+^ T cells responding specifically to a DENV-NS1 peptide by *in vivo* cytotoxicity assay [14]. In this analysis, they found that splenocytes isolated from naïve mice were not able to cause substantial cell lysis in spleen of immunized-challenged mice. In contrast, lysis increased consistently when transferred splenocytes were pulsed with the DENV-NS1 peptide.

In the circulation of recipient mice, from the total recovered-CFSE^+^ cells, 4.02% were CFSE^low^, what represented about 16 times the value found in samples from Naïve_transf_ group (0.25%). From these CFSE^low^ cells, 51.2% were CD4^+^ and 10.7% were CD8^+^ (Fig 5 panel C middle). This finding is consistent with the higher percentages of CD45RB^low^ T cells found in the circulation of vaccinated-challenged mice at the 7^th^ dpi. Data suggested that upon lymphocyte activation in secondary lymphoid organs, T cells migrated to the periphery, possibly to exert an effector role in sites committed by the infection.

Next, we aimed to identify the presence of DENV-specific T cells in infected organs. As we already knew that after i.c.-DENV challenge some virus particles can reach the liver [6], and that this organ is an important target during DENV infection in mice [6,47–51], this site was considered for further investigation. We found that liver samples of Vac_transf_ group presented 20.1% of CFSE^low^ cells (of which 28.9% were CD4^+^ and 8.89% were CD8^+^) out of the recovered-CFSE^+^ events. This represented a substantial increase of nearly 20 times, in comparison to the amount recovered from livers of Naïve_transf_ mice (1.25%) (Fig 5 panel C bottom). It is interesting to note that, in liver samples, the percentage of total CFSE^+^ cells was more than 4 times higher in control animals compared to vaccinated mice (4.09% and 0.45% in Naïve_transf_ and Vac_transf_ groups, respectively), while the percentage of CFSE^low^ cells in vaccinated group was significantly higher (23,1% comparing to 1.25% in control group). Hence, from results obtained in this CFSE-assisted cell tracking experiment we could infer that vaccination with pcTPANS1 would promote its protection, at least in part, by directing specific T cell migration to different target organs in the host.

In a brief conclusion, the experiment considered here could give us a more complete idea regarding the T cell response induced by pcTPANS1 vaccination upon i.c.-DENV infection. We found that DNA vaccination was able to induce T cell proliferation in spleen of infected hosts, followed by migration of lymphocytes to peripheral organs such as the liver, as CFSE^low^ T cells were consistently found in the circulation of these animals.

## Conclusion

In this work, we presented new findings concerning aspects of cellular immunity involved in the protection against dengue elicited by a DNA vaccine (pcTPANS1), which is based on the NS1 antigen. Experiments conducted using a traditional immunocompetent animal approach where BALB/c mice were i.c.-challenged with a mouse-brain adapted dengue virus. Apart from the drawbacks that this model present, such as the non-physiological infection route and the induced clinical manifestations that differ from the classical disease as in humans, it still brings important benefits for the investigation of the protection generated by vaccination. Besides the straightforward correlate of protection based on the prevention of morbidity, a main characteristic of this model is the immunocompetent environment. The latter is fundamental for the understanding of the immune mechanisms, since no restriction in its development or regulation is present.

From our study, we initially found that vaccinated-challenged mice exhibited increased levels of activated T cells in spleen and blood from the 5^th^-7^th^ dpi onwards. This fact indicated the existence of a systemic arrangement of immunity even after the development of infection from the immune-privileged site. Moreover, the surface expression of CD45RB^low^ on CD4^+^ and CD8^+^ T cells correlated with the absence of morbidity upon i.c. infection with DENV. Adoptive transference experiments revealed that NS1-specific T cells induced by vaccination proliferate and migrate to the liver of mice after virus challenge, indicating that T cells are targeted to affected peripheral organs in this animal model. Additionally, based on the reduced levels of pro-inflammatory cytokines detected in vaccinated animals, we suggest that the immune response elicited by pcTPANS1 occurs in a more controlled fashion upon virus challenge. After all, this study broadens the view about the T cell-mediated immunity concerning the pcTPANS1 DNA vaccine and the i.c. infection model of dengue. Ultimately, this work provided new insights and a more complete picture about the protective scenario yielded by vaccination under the above circumstances.

## Supporting Information

S1 Fig Calibration curves for cytokine assessment. The cytometric bead array (CBA) technique was implemented in order to measure the serum levels of IL-12p70, TNF-α, IFN-γ, MCP-1, IL-10 and IL-6 in samples collected from studied animals along the kinetics. Standard cytokines, provided by the manufacturer, were pooled and serially diluted for the construction of calibration curves. Cytokine levels (pg/ml) were derived using the FCAP array software (BD Bioscience) based on values of median fluorescence intensity (MFI) obtained from different dilutions, which are proportional to the quantity of a given standard cytokine in the sample. The 5-parameter logistic model (5PLM) was applied to obtain the regression curves. Coefficient of correlations (R^2^) are exhibited for each curve of analyzed cytokine.
(TIF)

S2 Fig Obtention and evaluation of CFSE-stained samples used for cell-transference experiments. Splenocytes obtained from non-stimulated (naïve) or pcTPANS1-vaccinated BALB/c mice (n = 5 for each group) were pooled and submitted to a T cell enrichment procedure using nylon wool column. Flow cytometry analysis were performed using samples before and after the enrichment procedure considering anti-CD3, anti-CD4 and anti-CD8 cell-surface markers simultaneously. In sequence, enriched T cell suspensions were labeled with CFSE and also evaluated by flow cytometry using anti-CD4 and anti-CD8. The figure shows original flow cytometry histograms presenting the percentage of CD3^+^ cells in splenocyte suspensions (A) before and (B) after the T cell enrichment procedure. The percentage of CD4^+^ and CD8^+^ cells are exhibited as *dot plot* representations. (C) Representative flow cytometry analysis of the CFSE-stained samples exhibiting its high fluorescence intensity on the FL1 (CFSE) channel. Percentages of CD4^+^ and CD8^+^ cells are also exhibited as *dot plot* representations.
(TIF)
