# Supplementary figures and images for: Aspects of T Cell-Mediated Immunity Induced in Mice by a DNA Vaccine Based on the Dengue-NS1 Antigen after Challenge by the Intracerebral Route
Source: PLoS One. 2016 Sep 15;11(9):e0163240. doi: 10.1371/journal.pone.0163240 (PMC5024998; doi:10.1371/journal.pone.0163240)

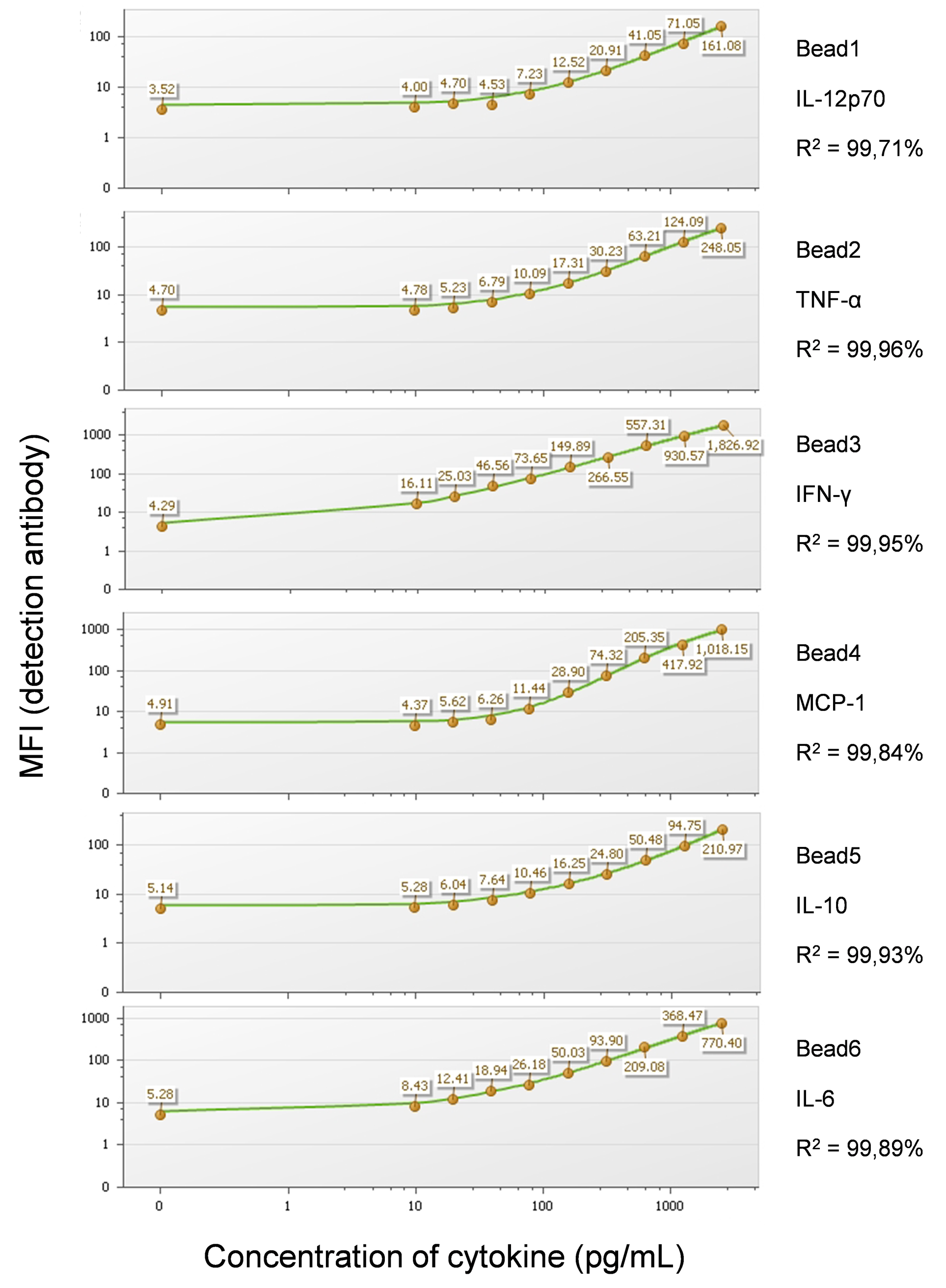

Supplement: S1 Fig — The cytometric bead array (CBA) technique was implemented in order to measure the serum levels of IL-12p70, TNF-α, IFN-γ, MCP-1, IL-10 and IL-6 in samples collected from studied animals along the kinetics. Standard cytokines, provided by the manufacturer, were pooled and serially diluted for the construction of calibration curves. Cytokine levels (pg/ml) were derived using the FCAP array software (BD Bioscience) based on values of median fluorescence intensity (MFI) obtained from different dilutions, which are proportional to the quantity of a given standard cytokine in the sample. The 5-parameter logistic model (5PLM) was applied to obtain the regression curves. Coefficient of correlations (R2) are exhibited for each curve of analyzed cytokine. (TIF) [file pone.0163240.s001.tif]

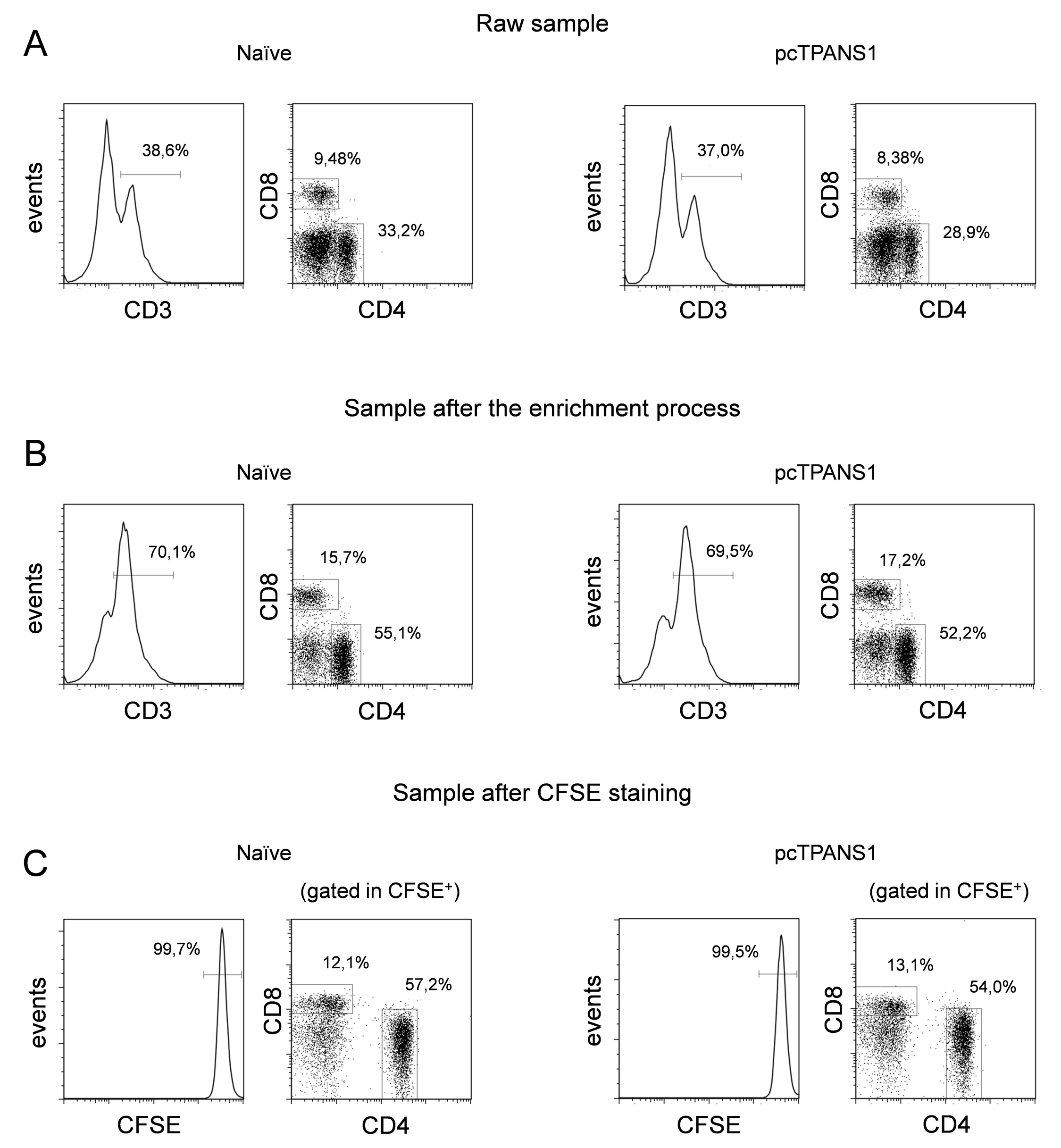

Supplement: S2 Fig — Splenocytes obtained from non-stimulated (naïve) or pcTPANS1-vaccinated BALB/c mice (n = 5 for each group) were pooled and submitted to a T cell enrichment procedure using nylon wool column. Flow cytometry analysis were performed using samples before and after the enrichment procedure considering anti-CD3, anti-CD4 and anti-CD8 cell-surface markers simultaneously. In sequence, enriched T cell suspensions were labeled with CFSE and also evaluated by flow cytometry using anti-CD4 and anti-CD8. The figure shows original flow cytometry histograms presenting the percentage of CD3+ cells in splenocyte suspensions (A) before and (B) after the T cell enrichment procedure. The percentage of CD4+ and CD8+ cells are exhibited as dot plot representations. (C) Representative flow cytometry analysis of the CFSE-stained samples exhibiting its high fluorescence intensity on the FL1 (CFSE) channel. Percentages of CD4+ and CD8+ cells are also exhibited as dot plot representations. (TIF) [file pone.0163240.s002.tif]
